# Testosterone differentially regulates targets of lipid and glucose metabolism in liver, muscle and adipose tissues of the testicular feminised mouse

**DOI:** 10.1007/s12020-016-1019-1

**Published:** 2016-08-04

**Authors:** Daniel M. Kelly, Samia Akhtar, Donna J. Sellers, Vakkat Muraleedharan, Kevin S. Channer, T. Hugh Jones

**Affiliations:** 1 Department of Oncology and Metabolism, Medical School, The University of Sheffield, Sheffield, UK; 2 Biomolecular Research Centre, Sheffield Hallam University, Sheffield, UK; 3 Centre for Diabetes and Endocrinology, Barnsley Hospital NHS Foundation Trust, Barnsley, UK; 4 Department of Cardiology, Royal Hallamshire Hospital, Sheffield, UK; 5 Present address: Faculty of Health Sciences and Medicine, Bond University, Gold Coast, Queensland 4229, Australia

**Keywords:** Type 2 diabetes, Metabolism, Testosterone, Androgen receptor, Adipose tissue

## Abstract

Testosterone deficiency is commonly associated with obesity, metabolic syndrome, type 2 diabetes and their clinical consequences—hepatic steatosis and atherosclerosis. The testicular feminised mouse (non-functional androgen receptor and low testosterone) develops fatty liver and aortic lipid streaks on a high-fat diet, whereas androgen-replete XY littermate controls do not. Testosterone treatment ameliorates these effects, although the underlying mechanisms remain unknown. We compared the influence of testosterone on the expression of regulatory targets of glucose, cholesterol and lipid metabolism in muscle, liver, abdominal subcutaneous and visceral adipose tissue. Testicular feminised mice displayed significantly reduced GLUT4 in muscle and glycolytic enzymes in muscle, liver and abdominal subcutaneous but not visceral adipose tissue. Lipoprotein lipase required for fatty acid uptake was only reduced in subcutaneous adipose tissue; enzymes of fatty acid synthesis were increased in liver and subcutaneous tissue. Stearoyl-CoA desaturase-1 that catalyses oleic acid synthesis and is associated with insulin resistance was increased in visceral adipose tissue and cholesterol efflux components (ABCA1, apoE) were decreased in subcutaneous and liver tissue. Master regulator nuclear receptors involved in metabolism—Liver X receptor expression was suppressed in all tissues except visceral adipose tissue, whereas PPARγ was lower in abdominal subcutaneous and visceral adipose tissue and PPARα only in abdominal subcutaneous. Testosterone treatment improved the expression (androgen receptor independent) of some targets but not all. These exploratory data suggest that androgen deficiency may reduce the buffering capability for glucose uptake and utilisation in abdominal subcutaneous and muscle and fatty acids in abdominal subcutaneous. This would lead to an overspill and uptake of excess glucose and triglycerides into visceral adipose tissue, liver and arterial walls.

## Introduction

Evidence suggests that testosterone deficiency in men is an independent cardiovascular risk factor which is associated with obesity, metabolic syndrome (MetS) and type-2 diabetes (T2D) [1, 2]. Insulin resistance, which is common to all of these conditions, results in diminished glucose utilisation and conversion of the excess glucose into fat. Higher circulating triglycerides then lead to an overspill of fat into ectopic storage in liver and arteries as well as increasing the accumulation of visceral fat. The degree of insulin resistance correlates negatively with serum testosterone [3, 4]. Although the causality of this relationship is often debated, growing evidence indicates testosterone is a metabolic multi-system player [5]. Epidemiological studies support a bidirectional relationship between serum testosterone and obesity which may be explained by the hypogonadal–obesity–adipocytokine hypothesis [6, 7]. Androgen deprivation therapy for the treatment of prostate cancer in men, whilst reducing tumour growth, also increases the risk of coronary heart disease, diabetes and cardiovascular death, indicating that testosterone deficiency may promote atherosclerosis [8, 9]. Some trials have reported that achieving a normal physiological testosterone concentration through the administration of testosterone replacement therapy (TRT) improves vascular function and risk factors for atherosclerosis, including reducing central adiposity, percentage body fat, fatty liver and insulin resistance, and improving lipid profiles insulin sensitivity and inflammatory profiles [2, 10–15].

A limited number of in vivo and in vitro investigations have highlighted potential molecular targets of testosterone action in metabolic regulation, although a detailed analysis of tissue-specific actions remains absent from the literature [2]. We have previously reported that low testosterone in the testicular feminised (Tfm) mouse (which displays very low testosterone levels and non-functional androgen receptors) is associated with increased lipid deposition in the aortic root and liver when mice are fed a high-cholesterol diet [16–18]. Testosterone treatment to return levels to those seen in wild-type counterparts significantly reduced aortic fatty steaks and hepatic lipid accumulation with an associated reduction in de novo lipogenesis in the liver in Tfm mice [17].

While a growing body of evidence points towards the presence of heterogeneity regarding insulin responsiveness and lipid homeostasis among different tissues [19], the mechanisms by which testosterone may impart beneficial actions on insulin sensitivity and hence the development of MetS, T2D and cardiovascular risk remain unknown but are likely to be tissue dependent and involve multiple targets of lipid and carbohydrate metabolism. In the present exploratory study, we aim to investigate whether the metabolic protective effects of testosterone act via modulation of the expression of key targets involved in lipid and glucose metabolism in muscle, liver and adipose tissue of cholesterol-fed Tfm mice. Specifically, we investigate key regulatory enzymes of glycolysis, glycogen synthesis, pentose phosphate pathway, glucose transporters, fatty acid synthesis, fatty acid uptake, cholesterol synthesis and efflux, and master regulators of metabolism (see Table 1).

**Table 1:** Qiagen qPCR primers

Target	Gene	Function	Product ref.
Fatty acid synthase	*Fasn*	Catalyses the formation of long-chain fatty acids in fatty acid synthesis	QT00149240
Acetyl coA carboxylase	*Acaca*	Essential role in regulating fatty acid synthesis	QT01554441
Stearoyl-CoA desaturase 1	*Scd1*	Catalyses a rate-limiting step in the synthesis of unsaturated fatty acids. Key enzyme in fatty acid metabolism.	QT00291151
Lipoprotein lipase	*Lpl*	Hydrolysis of triglycerides into free fatty acids	QT01750469
Hormone sensitive lipase	*Lipe*	Hydrolyses stored triglycerides to free fatty acids	QT00169057
3-hydroxy-3-methylglutaryl-CoA reductase	*Hmgcr*	Rate-controlling enzyme of the mevalonate pathway that produces cholesterol	QT01037848
Sterol regulatory element-binding protein 1	*Srebf1*	Cholesterol biosynthesis and uptake, and fatty acid biosynthesis	QT00167055
Sterol regulatory element-binding protein 2	*Srebf2*	Cholesterol biosynthesis and uptake, and fatty acid biosynthesis	QT01045870
Apolipoprotein E	*Apoe*	Lipoprotein metabolism and transport.	QT01043889
ATP-binding cassette transporter A1	*Abca1*	Major regulator of cellular cholesterol efflux and phospholipid homoeostasis	QT00165690
ATP-binding cassette transporter G5	*Abcg5*	Cellular cholesterol efflux, promote biliary excretion of sterols.	QT00157752
Insulin receptor substrate 1	*Irs1*	Transmitting signals from the insulin and insulin-like growth factor-1 (IGF-1) receptors to intracellular pathways in insulin signalling	QT00251657
Hexokinase 2	*Hk2*	Phosphorylates glucose to glucose 6-phosphate in the glycolytic pathway	QT00155582
Hexokinase 4 (Glucokinase)	*Gck*	Phosphorylates glucose to glucose 6-phosphate in the glycolytic pathway	QT00140007
Phosphofructokinase	*Pfk*	Converts fructose-6-phosphate to fructose-1,6-bisphosphate, one of the most important regulatory enzymes of glycolysis	QT00159754
Carbohydrate-responsive element-binding protein	*Chrebp*	Activates of several regulatory enzymes of glycolysis and lipogenesis	QT00125335
Glucose transporter 4	*Glut4*	Cellular glucose transport	QT01044946
Glucose-6-phosphate dehydrogenase	*G6pdx*	Enzyme in the pentose phosphate pathway, often for tissues actively engaged in biosynthesis of fatty acids	QT01748957
Glycogen synthase	*Gys*	Converts glucose to glycogen for storage, regulating glycogen/glucose levels	QT00162099
Liver X receptor alpha	*Nr1h3*	Nuclear receptor transcription factor regulating cholesterol, fatty acid, and glucose homoeostasis	QT00113729
Peroxisome proliferator-activated receptor alpha	*Ppara*	Transcription factor and major regulator of lipid metabolism	QT00137984
Peroxisome proliferator-activated receptor gamma	*Pparg*	Regulates fatty acid storage and glucose metabolism	QT00100296
Beta 2 microglobulin	*B2m*	Reference gene	QT01149547
Glyceraldehyde 3-phosphate dehydrogenase	*Gapdh*	Reference gene	QT01658692

## Materials and methods

### Animals

The Tfm mouse was used as a model of testosterone deficiency and androgen receptor (AR) dysfunction as previously described [16–18]. The loss of 17α-hydroxylase, a key enzyme necessary for testosterone synthesis, leads to serum levels of testosterone in the Tfm mouse that are severely (approximately 10-fold) reduced compared to normal XY littermate controls [20, 21]. In addition, a natural mutation in the gene encoding the AR leads to the formation of a truncated receptor protein which lacks both DNA-binding and steroid-binding domains, rendering it non-functional [22, 23]. This model therefore allows potential AR-dependent and independent effects to be investigated. All procedures were carried out under the jurisdiction of a UK Home Office project licence, governed by the UK Animals Scientific Procedures Act 1986. Mice were bred as previously described [20]. Animal numbers were calculated based on our previous investigation [16] for a significance level of 5 %, and a power of 90 % for the primary outcome measure of lipid deposition in the aortic root (see [18]). Where available, preliminary data was used for calculation of sample numbers of individual variables.

### Experimental design and tissue collection

Eight-week-old Tfm and XY littermate mice were fed a high-fat, high-cholesterol diet, containing 42 % butterfat, 1.25 % cholesterol and 0.5 % cholate (Special Diet Services, Essex, UK) ad libitum for a period of 28 weeks. Separate 7-week-old Tfm mice were first randomly assigned to one of two groups: a placebo group receiving a once-fortnightly intramuscular injection of 10 μL of saline (*n* = 14), or testosterone group (*n* = 14) receiving a once-fortnightly intramuscular injection of 10 μL of 100 mg/mL testosterone esters (Sustanon100; testosterone propionate 20 mg/mL, testosterone phenylpropionate 40 mg/mL and testosterone isocaproate 40 mg/mL, Organon Laboratories Ltd, Cambridge, UK), providing a dose of 50 mg/kg, previously shown to replace circulating levels to those of wild-type littermate mice [16]. XY littermate mice (*n* = 14) received placebo injections (10 μL saline). Animals were caged under standard conditions in a temperature and humidity-controlled room on a 12 -h light:12-h darkness cycle. Water and food were unrestricted throughout the study.

At the end of the experimental period, which corresponded with the midway point of the fortnightly injection cycle, whole blood was collected from the thoracic cavity following mid-line sternotomy and severance of the thoracic aorta. Following centrifugation, serum samples were frozen at −80 °C. The liver was removed from the abdomen, skeletal muscle dissected from the quadriceps of the hind legs and fat tissue collected from subcutaneous and visceral abdominal regions. The heart with thoracic aorta attached was carefully dissected free from the adventitia and perfused. Tissues were processed for both histological and gene and protein expression analysis and were archived for future analysis. Analyses were made on individual samples.

### Measurement of total testosterone and 17β-estradiol

Serum quantification of total testosterone (DRG Instruments GmBH, Marburg, Germany) and 17β-estradiol (Demeditec Diagnostics, Kiel, Germany) was measured in duplicate via ELISA (measurement range 0.2–16 ng/mL and 3–200 pg/mL, respectively).

### Quantitative analysis of mRNA

Total RNA was extracted from approximately 100 mg of snap-frozen tissue, reverse transcribed and cDNA (2 μL) used for qPCR, using commercial SYBR green reagents (Qiagen) as described previously [17]. Primers were purchased pre-validated (QuantiTech primer assays; Qiagen), with specified amplification efficiencies of 100 % (±10 %) (see Table 1). Primers for Β-2 microglobulin (*B2m*) were also included and served as an internal reference control, selected as the most stable gene from a panel of commonly used reference genes (*Gapdh*, beta-actin, ribosomal protein 13A). Each reaction was carried out in triplicate with cycling and detection of fluorescent signal carried out using an Agilent Mx3000P QPCR System. Results were corrected for the expression of the house-keeping gene and normalised to the XY littermates as a control. Relative copy number was expressed as fold change 2-(ddCT).

### Western immunoblotting

In this exploratory study we selected targets that were significantly altered at the gene expression level for analysis by western blotting. Due to low concentrations of protein ascertainable from limited availability of adipose tissue, western blotting was unable to be carried out on subcutaneous and visceral samples. Protein was extracted from 200 mg of mouse liver or muscle tissue as previously described [17]. In brief, 50 µg of total isolated protein was separated by electrophoresis and transferred to nitrocellulose membranes (BioRad, Hertfordshire, UK). Membranes were blocked for 1 h in 5 % dried semi-skimmed milk diluted in tris/glycine (TG) buffer containing 0.05 % Tween 20 (TGT; BioRad, UK). Primary antibodies were incubated overnight at 4 °C diluted in either 5% bovine serum albumin/TGT, 5 % milk/TGT or 2.5 % milk/BSA (see Table 2). Immunoreactive proteins were detected using anti-rabbit IgG HRP-linked secondary antibody (1:500, Cell Signalling) for polyclonal antibody detection or anti-mouse IgG HRP-linked secondary antibody (1:500, Cell Signalling) followed by a chemiluminescence peroxidase substrate kit (Roche, Sussex, UK). Band intensities were quantified using Genetools software (Syngene, Cambridge, UK) relative to the house-keeping protein GAPDH or Calnexin.

**Table 2:** Antibody parameters

Antibody	Concentration	Diluent	Supplier
FASN	1:500	2.5 % milk bsa in tbs	CST
ACACA	1:500	2.5 % milk bsa in tbs	CST
ABCA1	1:250	2.5 % milk bsa in tbs	abcam
APOE	1:250	5 % milk in tbs	abcam
GCK	1:500	5 % milk in tbs	abcam
PFK	1:250	0.01 % milk bsa in tbs	Proteintech
GLUT4	1:500	2.5 % milk bsa in tbs	CST
HK2	1:500	2.5 % milk bsa in tbs	CST
LXR	1:500	1 % milk bsa in tbs	abcam
G6PD	1:500	0.01 % milk bsa in tbs	Sigma
GAPDH	1:5000	2.5 % milk bsa in tbs	abcam
Calnexin	1:1000	5 % milk in tbs	CST

*bsa* bovine serum albumin, *tbs* tris-buffered saline, *CST* cell signalling technologies

### Statistical analysis

Results are presented as mean ± SEM. Differences between groups with normally distributed data were compared using unpaired *t* tests without assuming consistent standard deviations of groups. Mann–Whitney *U* tests were used where data did not follow a normal distribution. Corrections for multiple comparisons were made using the Sidak-Bonferroni post hoc test. Significance was accepted at *p* ≤ 0.05.

## Results

**Fig. 1:**
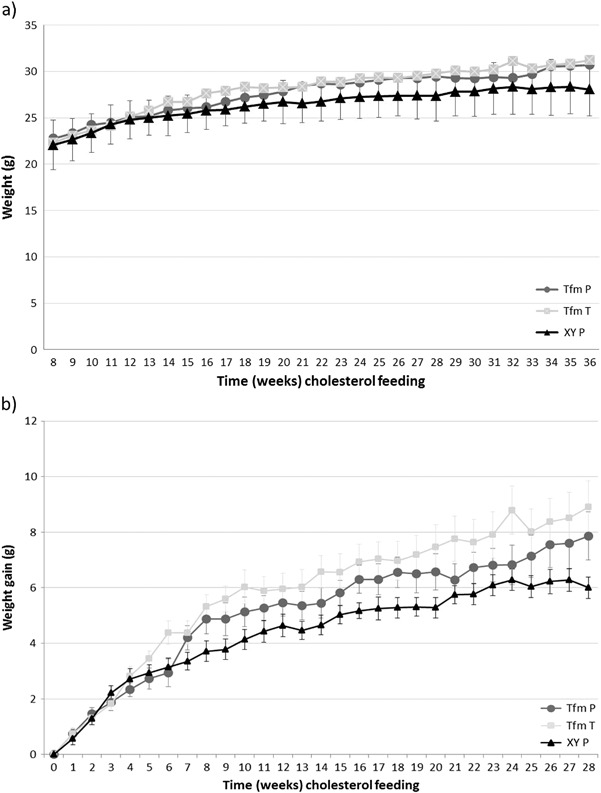
Animal weights and weight gain. Tfm mice receiving either placebo (Tfm P) or testosterone (Tfm T) and wild-type XY littermates receiving placebo (XY P) had total body weight (**a**) measured at weekly intervals from the commencement of high-cholesterol diet feeding at week 8 through to the end of the study at week 36. Weight gain (**b**) was calculated from starting weights of individual animals. No significant differences were noted between groups

Serum testosterone levels were greatly reduced in Tfm mice (2.2 ± 1.2 nM, *p* = 0.03) compared to wild-type equivalents (16.5 ± 4.3 nM).^1^ Testosterone treatment of Tfm mice increased serum levels of testosterone comparable to wild-type levels (14.7 ± 5.2 nM, *p* = 0.98). 17-β estradiol levels were similar between all groups, Tfm mice (94.2±15.5 pmol) compared to wild-type (106.0 ± 33.9 pmol, *p* = 0.17) and testosterone-treated Tfm mice (135.2 ± 28.7 pmol, *p* = 0.99). Animal weights and weight gain did not significantly differ between groups over the duration of the 28 week feeding period but there was a trend towards Tfm mice gaining more weight compared to littermates by the end of the study period (*p* = 0.066, *n* = 14; Fig. 1).

### Carbohydrate metabolism

Gene expression of the glycolytic regulatory gateway enzymes hexokinase (*Hk2*, *Gck*) and *Pfk* was significantly lower in muscle (*p* = 0.012, *p* = 0.032), liver (*p* = 0.002, *p* = 0.04) and SAT (*p* = 0.009, *p* = 0.03) but not in VAT of Tfm-placebo mice compared to XY littermates (Table 3). Testosterone administration increased *Gck* expression (*p* = 0.015) in the liver of Tfm mice but these enzymes were not significantly altered in other tissues by treatment. *Glut4* was similarly decreased in muscle (*p* = 0.015) and SAT (*p* = 0.014) of Tfm mice versus wild-type mice, with no effect of testosterone treatment. Hepatic *G6pdx* was elevated in Tfm mice compared to XY mice (*p* < 0.001) and testosterone treatment showed a trend to reducing this expression in Tfm mice (*p* = 0.056). All other gene targets were not altered between experimental groups in the tissues investigated.

**Table 3:** Gene expression of targets of lipid and glucose regulation in muscle, liver, subcutaneous and visceral adipose tissue of Tfm mice

Fat Metabolism													
		Muscle			Liver			Subcutaneous Fat			Visceral Fat		
Gene	Symbol	XY-P	Tfm-P	Tfm-S100	XY-P	Tfm-P	Tfm-S100	XY-P	Tfm-P	Tfm-S100	XY-P	Tfm-P	Tfm-S100
Acetyl CoA carboxylase alpha	ACACA	1.28 ± 0.27	1.65 ± 0.46	0.77 ± 0.14	1.09 ± 0.13	**2.49** ± 0.64*	1.30 ± 0.29	1.22 ± 0.28	1.15 ± 0.36	1.89 ± 0.62	1.07 ± 0.16	1.02 ± 0.27	0.95 ± 0.35
Fatty acid synthase	FASN	1.52 ± 0.43	1.48 ± 0.56	0.56 ± 0.14	1.15 ± 0.17	**11.42** ± 4.93*	2.99 ± 0.88	1.20 ± 0.28	2.89 ± 1.49	4.76 ± 1.90	1.12 ± 0.23	1.35 ± 0.36	0.87 ± 0.30
Stearoyl-CoA desaturase-1	SCD1	1.06 ± 0.11	1.30 ± 0.24	1.37 ± 0.29	1.04 ± 0.14	2.45 ± 1.15	1.38 ± 0.15	1.2 ± 0.29	3.05 ± 1.37	0.70 ± 0.15	1.10 ± 0.23	**4.99** ± 1.64*	**0.94** ± **0.15** ^***†***^
Lipoprotein lipase	LPL	1.24 ± 0.31	0.90 ± 0.30	0.83 ± 0.20	1.07 ± 0.13	1.26 ± 0.45	0.90 ± 0.26	1.03 ± 0.08	**0.70** ± 0.09*	0.81 ± 0.22	1.08 ± 0.13	0.99 ± 0.20	**2.18** ± **0.36** ^***†***^
Hormone sensitive lipase	Lipe	1.20 ± 0.46	1.25 ± 0.24	1.18 ± 0.49	1.03 ± 0.08	1.24 ± 0.16	0.85 ± 0.10	1.17 ± 0.17	1.49 ± 0.29	1.14 ± 0.20	1.05 ± 0.14	1.10 ± 0.27	0.82 ± 0.21

Cholesterol Homeostasis													
		Muscle			Liver			Subcutaneous Fat			Visceral Fat		
Gene	Symbol	XY-P	Tfm-P	Tfm-S100	XY-P	Tfm-P	Tfm-S100	XY-P	Tfm-P	Tfm-S100	XY-P	Tfm-P	Tfm-S100
3-hydroxy-3-methyl-glutaryl-CoA reductase	HMGCoAr	_	_	_	1.06 ± 0.11	3.19 ± 2.09	1.15 ± 0.18	1.12 ± 0.19	0.88 ± 0.16	1.33 ± 0.37	_	_	_
Sterol Regulatory Element-Binding Protein	SREBPf1	1.29 ± 0.26	0.74 ± 0.21	1.47 ± 0.39	1.13 ± 0.16	1.01 ± 0.19	1.32 ± 0.29	1.07 ± 0.17	**0.36** ± 0.09**	**0.86** ± **0.16** ^***†***^	0.81 ± 0.13	0.59 ± 0.25	0.78 ± 0.13
Sterol Regulatory Element-Binding Protein	SREBPf2	1.17 ± 0.21	0.80 ± 0.28	1.99 ± 0.62	1.08 ± 0.13	1.44 ± 0.22	1.17 ± 0.16	1.07 ± 0.17	**0.42** ± 0.08**	**1.12** ± **0.33** ^***c***^	0.80 ± 0.14	0.56 ± 0.21	0.79 ± 0.13
Apolipoprotein E	ApoE	_	_	_	1.07 ± 0.05	**0.77** ± 0.09**	**1.14** ± **0.11** ^***†***^	1.02 ± 0.08	**0.59** ± 0.13*	**1.14** ± **0.16** ^***†***^	1.03 ± 0.10	0.75 ± 0.16	0.87 ± 0.06
ATP-binding cassette transporter A1	ABCA1	_	_	_	1.05 ± 0.04	**0.71** ± 0.09**	**1.10** ± **0.13** ^***†***^	1.07 ± 0.14	0.77 ± 0.12	0.63 ± 0.11	1.14 ± 0.24	0.98 ± 0.20	1.19 ± 0.32
ATP-binding cassette transporter G5	ABCG5	_	_	_	1.01 ± 0.06	0.81 ± 0.15	1.01 ± 0.08	_	_	_	_	_	_

Carbohydrate Metabolism													
		Muscle			Liver			Subcutaneous Fat			Visceral Fat		
Gene	Symbol	XY-P	Tfm-P	Tfm-S100	XY-P	Tfm-P	Tfm-S100	XY-P	Tfm-P	Tfm-S100	XY-P	Tfm-P	Tfm-S100
Insulin receptor substrate 1	IRS1	1.34 ± 0.32	1.37 ± 0.59	1.85 ± 0.94	1.08 ± 0.17	1.74 ± 0.54	1.13 ± 0.29	_	_	_	_	_	_
Hexokinase 2	HK2	1.18 ± 0.19	**0.50** ± 0.16*	0.54 ± 0.10	_	_	_	1.32 ± 0.36	**0.24** ± 0.05**	**0.61** ± **0.19** ^***†***^	0.97 ± 0.23	1.00 ± 0.21	1.57 ± 0.54
Hexokinase 4 (Glucokinase)	GCK	_	_	_	1.07 ± 0.10	**0.47** ± 0.14**	**0.97** ± **0.13** ^***†***^	_	_	_	_	_	_
Phosphofructokinase	PFK	1.28 ± 0.23	**0.64** ± 0.16*	0.62 ± 0.10	1.19 ± 0.11	**0.79** ± 0.15*	0.77 ± 0.06	1.76 ± 0.68	**0.16** ± 0.05*	0.54 ± 0.31	_	_	_
mitogen-activated protein kinase kinase 1	MAP2K1	1.22 ± 0.22	**0.65** ± **0.19** ^***a***^	1.09 ± 0.21	1.09 ± 0.14	1.01 ± 0.14	**1.62** ± **0.21** ^***†***^	_	_	_	_	_	_
Carbohydrate regulatory element binding protein	ChREBP	1.25 ± 0.24	2.16 ± 1.02	1.16 ± 0.22	1.05 ± 0.12	1.17 ± 0.13	1.11 ± 0.13	1.00 ± 0.19	1.76 ± 0.47	1.53 ± 0.38	1.33 ± 0.38	1.96 ± 0.7	1.76 ± 0.37
Glucose transporter 4	GLUT4	1.20 ± 0.19	**0.59** ± 0.14*	0.71 ± 0.13	_	_	_	1.31 ± 0.32	**0.37** ± 0.12*	0.73 ± 0.29	1.03 ± 0.10	1.24 ± 0.26	0.83 ± 0.16
glucose-6-phosphate 1-dehydrogenase X	G6PDx	1.13 ± 0.19	1.72 ± 0.38	0.92 ± 0.12	1.03 ± 0.07	**1.99** ± 0.20***	**1.45** ± **0.18** ^***b***^	1.06 ± 0.23	2.67 ± 1.36	1.09 ± 0.16	1.57 ± 0.59	2.53 ± 0.87	1.69 ± 0.4
Glycogen synthase	Gys1	1.14 ± 0.15	1.01 ± 0.31	1.82 ± 0.72	1.18 ± 0.18	2.13 ± 0.65	1.56 ± 0.22	_	_	_	_	_	_

Master Regulators													
		Muscle			Liver			Subcutaneous Fat			Visceral Fat		
Gene	Symbol	XY-P	Tfm-P	Tfm-S100	XY-P	Tfm-P	Tfm-S100	XY-P	Tfm-P	Tfm-S100	XY-P	Tfm-P	Tfm-S100
Liver X receptor	LXR	1.35 ± 0.29	**0.62** ± 0.12*	**1.50** ± **0.27** ^***††***^	1.05 ± 0.09	**0.66** ± 0.08**	**1.28** ± **0.24** ^***†***^	1.07 ± 0.14	**0.42** ± 0.13**	**1.15** ± **0.28** ^***†***^	1.27 ± 0.35	1.00 ± 0.30	1.44 ± 0.48
Peroxisome proliferator-activated receptor alpha	PPARa	1.16 ± 0.20	1.65 ± 0.78	0.94 ± 0.21	1.02 ± 0.07	1.84 ± 0.55	0.93 ± 0.14	1.13 ± 0.23	0.41 ± 0.11	0.69 ± 0.22	1.09 ± 0.20	0.92 ± 0.32	0.98 ± 0.22
Peroxisome proliferator-activated receptor gamma	PPARg	1.29 ± 0.31	1.29 ± 0.62	0.63 ± 0.17	1.09 ± 0.14	0.99 ± 0.19	1.29 ± 0.23	1.06 ± 0.14	0.67 ± 0.06	0.82 ± 0.12	1.05 ± 0.11	**0.49** ± 0.08***	0.66 ± 0.10

Relative tissue-specific qPCR end-point analysis of selected genes of (**a**) fat metabolism, (**b**) cholesterol homeostasis, (**c**) carbohydrate metabolism and (**d**) master regulators between Tfm placebo-treated versus XY littermates placebo-treated, and Tfm placebo-treated versus Tfm testosterone-treated. N=11. *p < 0.05, **p < 0.01, ***p < 0.001 versus XY placebo, ^†^p < 0.05, ^††^p < 0.01 versus Tfm placebo, ^a^p = 0.053, ^b^p^ =^ 0.058, ^c^p = 0.056.

**Fig. 2:**
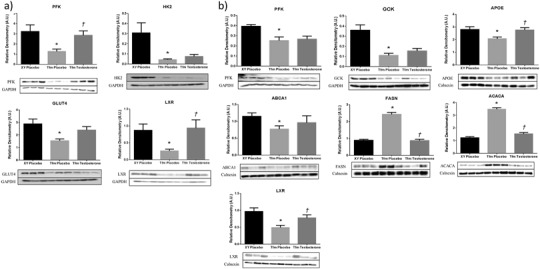
Protein expression of selected targets of lipid and glucose regulation in muscle and liver of Tfm mice. Semi quantitative western blot analysis in (**a**) muscle and (**b**) liver of Tfm mice receiving either placebo or testosterone and wild-type XY littermates receiving placebo at the end of the study period. Data are presented as densitometry arbitrary units and representative blot images. *N* = 6. *p < 0.05 versus XY placebo, ^†^ *p* < 0.05 versus Tfm placebo

Protein expression of PFK in the liver of experimental animals matched gene expression data with reduced levels in Tfm placebo mice compared to wild-type (*p* = 0.005) and no effect of treatment with testosterone (Fig. 2). Muscle protein expression of PFK was reduced in Tfm mice (*p* = 0.018) with a significant increase in expression following treatment (*p* = 0.01). Hepatic GCK protein was also reduced in Tfm mice receiving placebo (*p* = 0.001) as demonstrated at the gene level; however, testosterone administration had no effect showing discrepancy between gene and protein expression. HK2 in muscle was also reduced at the protein level in Tfm mice (*p* = 0.024), but there was no effect due to treatment. Muscle GLUT4 was decreased in Tfm mice compared to wildtype (*p* = 0.037) and testosterone administration demonstrated a trend towards increasing this expression (*p* = 0.053). We were unable to detect G6PD protein expression in the liver of experimental animals.

### Lipid metabolism

#### Cholesterol metabolism

Expression of cholesterol transporters, *Apoe* and *Abca1*, were reduced in the liver of Tfm mice compared to littermates (*p* = 0.009, *p* = 0.002). Treatment with testosterone significantly increased this expression (*p* = 0.027, *p* = 0.02), similar to wild-type levels (Table 3). Similarly, *Apoe* was decreased in SAT of Tfm mice (*p* = 0.01), an effect that was abolished by testosterone administration (*p* = 0.015 versus Tfm P). *Srebf1* and *Srebf2* expression was significantly lower in the SAT of Tfm mice versus XY littermates (*p* = 0.002, *p* = 0.003). Treatment with testosterone elevated these expression levels of *Srebf1* (*p* = 0.015) similar to those demonstrated in wild-type mice although not significantly so for *Srebf2* with only a trend towards increased expression observed (*p* = 0.053). In support of gene expression data, ABCA1 protein was significantly reduced in livers of Tfm mice compared to littermates and testosterone treated Tfms (Fig. 2). Hepatic APOE protein expression matched gene expression data with significantly lower levels in placebo-treated Tfm mice compared to XY littermates and testosterone-treated Tfm mice (*p* = 0.011, *p* = 0.007, respectively).

#### Fatty acid metabolism

Visceral adipose *Scd1* expression was significantly higher in Tfm mice receiving placebo than in XY littermates also receiving placebo injections (*p* = 0.034, Table 3). Testosterone treatment of Tfm mice returned expression levels to those of XY mice with a significant reduction compared to placebo-treated Tfm mice (*p* = 0.027). *t* test analysis similarly revealed an increase in hepatic *Scd1* expression in Tfm placebo mice although not statistically significant (*p* = 0.08). Decreased *Lpl* expression was observed in SAT from Tfm mice compared to wildtype (*p* = 0.016) although testosterone administration to Tfm animals had no effect on this. Hepatic gene expression of *Fasn* and *Acaca*, the key regulatory enzymes in de novo lipogenesis, were significantly increased in Tfm mice receiving placebo injections compared to wild-type littermates (*p* = 0.049, *p* = 0.042, respectively).^2^ Testosterone treatment decreased this expression but not significantly. Gene expression of all other lipid metabolism targets in liver and adipose tissue were not significantly different between animal groups. Western blotting showed hepatic protein expression of FASN and ACACA to be increased in Tfm mice confirming gene expression findings.^2^ Testosterone treatment significantly reduced the protein expression of these enzymes versus placebo treated Tfm mice to similar levels as XY littermates.

No targets of fat metabolism and cholesterol homeostasis displayed altered gene expression in muscle tissue from the different experimental groups.

### Master regulators

Gene expression of *Lxr* was significantly reduced in Tfm placebo mice in all tissues other than visceral adipose (muscle *p* = 0.032, liver *p* < 0.001, SAT *p* = 0.003), and testosterone administration increased expression significantly and back to wild-type levels in these tissues (muscle *p* = 0.008, liver *p* = 0.024, SAT *p* = 0.03). *Ppara* and *Pparg* were significantly reduced in SAT of Tfm mice receiving placebo versus XY littermate controls (*p* = 0.01, *p* = 0.02, respectively). Pparg was also reduced in visceral adipose tissue of Tfm mice (*p* = 0.001). Testosterone treatment had no effect on the altered expression of *Ppars* when compared to placebo treated Tfm mice (see Table 3).

LXR protein expression in liver and muscle demonstrated the same pattern indicated by gene expression analysis with a reduction in Tfm placebo mice compared to wild-type littermates (*p* = 0.001, *p* = 0.01). Treatment with testosterone elevated LXR levels significantly in liver and muscle (*p* = 0.024, *p* = 0.022), to similar levels seen in placebo-treated Tfm mice (Fig. 2).

## Discussion

Exploratory evidence from this study suggests that testosterone has tissue-specific metabolic effects in the regulation of gene targets which control glucose utilisation in liver, SAT and skeletal muscle, and lipid metabolism in liver and SAT. Some of these effects are, at least in part, androgen receptor-independent and may potentially explain some of the observed clinical benefit of testosterone in men with T2D and MetS.

### Testosterone effects on expression of targets of glucose metabolism

GLUT4 expression is known to correlate positively with insulin responsiveness and defects in expression of GLUT4 have been observed in patients with T2D [24]. We have shown that there is decreased expression of GLUT4 in muscle and SAT in the testosterone deficient Tfm mouse. Testosterone has previously been shown to increase the expression of GLUT4 in cultured skeletal muscle cells, hepatocytes and adipocytes [25–27] as well as augmenting membrane translocation and promoting glucose uptake in adipose and skeletal muscle tissue [27]. Key enzymes involved in glycolysis, PFK and HK, were significantly reduced in muscle, liver and SAT of Tfm mice. This supports previous studies which have demonstrated an increase in the activity of PFK and HK in cultured rat skeletal muscle cells and increased hexokinase activity in muscle tissue of castrated rats following testosterone treatment thus diminishing the raised blood levels of glucose seen in untreated control rats [27–29]. Improved glucose utilisation in muscle, liver and SAT by testosterone may reduce the conversion of glucose to fat in times of excess and improve insulin sensitivity thus reducing lipid accumulation in these and other tissues. This clinically would be very important in muscle as this tissue accounts for approximately 75 % of whole-body insulin-stimulated glucose uptake [30, 31].

We have also demonstrated in this study that the mRNA expression of Glucose-6-phosphate dehydrogenase (*G6pd*), the gateway enzyme in the pentose phosphate shunt pathway, is elevated in the liver of Tfm mice suggesting that glucose may also be utilised down this route during testosterone deficiency. NADPH is produced by G6PD in the pentose phosphate pathway supplying reducing power to contribute to fatty acid synthesis [32]. An aberrant increase of G6PD expression is present in obese and diabetic subjects, and overexpression of G6PD alters lipid metabolism, impairs insulin signalling and suppresses insulin-dependent glucose uptake in mouse adipocytes [32]. However, the exact role of hepatic G6PD in metabolic function is unknown.

### Testosterone effects on expression of targets of lipid metabolism

In the present study we demonstrate that testosterone deficiency negatively alters the expression of targets of lipid metabolism primarily in liver and SAT but had little effect in VAT. Decreased *Lpl* in Tfm mice with low testosterone may limit the hydrolysis of lipoproteins and the subsequent uptake of FFA into SAT. A previous study, however, has shown the expression of hormone sensitive lipase and LPL to be elevated in SAT of male mice with a selective adipocyte AR knockdown (fARKO) [33]. These mice were fed a normal chow diet and therefore LPL increase in the absence of testosterone activated AR signalling may reflect elevated subcutaneous lipid storage and decreased triglyceride usage as an energy source in other tissues in times of low fat intake. Treatment of hypogonadal men with TRT for 9 months resulted in a marked decrease in both LPL activity and triglyceride uptake in abdominal adipose tissue [34]. Following further investigation, although LPL expression or activity was not reported, the inhibition of lipid uptake after testosterone administration was apparent in visceral (omental plus mesenteric) and retroperitoneal but increased in abdominal SAT suggesting that inhibition of triglyceride assimilation may direct lipid to subcutaneous fat in TRT-treated men and may therefore involve altered lipase activity or expression in specific tissues [35], as suggested in the present study.

Human SCD1 is a critical control point of lipid partitioning with high SCD activity favouring fat storage and suppression of the enzyme activating metabolic pathways that promote the burning of fat and decrease lipid synthesis [36]. Mice with a targeted disruption of the *Scd1* gene have very low levels of VLDL and impaired triglyceride and cholesterol ester biosynthesis, as well as markedly reduced adiposity and decreased hepatic steatosis on both lean and ob / ob background despite higher food intake [37, 38]. In the present study we demonstrate significantly increased *Scd1* expression in VAT of Tfm mice and a trend towards increased expression in the liver. Beyond its role in fatty acid biosynthesis, SCD1 is an important factor in the pathogenesis of lipid-induced insulin resistance with SCD1 deficiency up-regulating insulin-signalling components and glycogen metabolism in insulin-sensitive tissues [38]. This suggests that testosterone has the potential to improve both lipid and glucose metabolism via reducing Scd1 expression in VAT and the liver of Tfm mice.

Lower subcutaneous *Apoe* expression in testosterone deficient Tfm mice may be indicative of decreased reverse cholesterol transport delivery of lipoproteins and cholesterol from SAT to the liver for clearance. This difference was not apparent in VAT supporting an important depot-specific role of APOE in adipose tissue substrate flux and accumulation of triglyceride in these depots [39]. Additionally, in the present study we demonstrate that mRNA expression of *Srebf1* and *Srebf2*, key transcription factors and master regulators of lipogenesis [40], were significantly decreased in SAT of Tfm mice compared to testosterone treated animals and wild-type controls. Similarly, orchidectomy significantly reduced hepatic SREBP-1 expression in mice fed a high fat diet or normal chow, an effect that was ameliorated by testosterone treatment in high fat diet conditions [41]. As SREBPs are known to directly induce transcription of many genes needed for uptake and synthesis of cholesterol, fatty acids, triglycerides and phospholipids [42]; taken together, these data lead us to hypothesise that testosterone deficiency may diminish SAT metabolic function and reduce lipid storage capacity.

Increased liver fat in Tfm mice from the present study is considered partly due to increased de novo lipogenesis and the expression of FASN and ACACA [17], which supported earlier studies indicating that a lack of testosterone action results in hepatic lipid accumulation [41–43]. The present study additionally indicates that ABCA1 and APOE, involved in cholesterol and lipoprotein efflux, are reduced in the testosterone-deficient state in the liver of Tfm mice. The overexpression of hepatic *Abca1* in transgenic mice results in a marked increase in HDL release, decreased LDL and significantly reduced atherosclerosis when compared with control mice [44]. Furthermore, increased hepatic cholesterol content was reported in these mice as the level of expression of the ABCA1 transporter decreased [45]. Indeed, Tfm mice from the present study have elevated total cholesterol and LDL compared to wild-type mice [18]. Therefore, the increased hepatic lipid accumulation in our Tfm mice may additionally result from absence of beneficial testosterone effects on lipid transport.

### Testosterone effects on master regulators of lipid and glucose metabolism

Testosterone altered the expression of master metabolic regulators as a potential signalling mode of action to influence lipid and glucose regulation. Reduced expression of the nuclear receptor, liver X receptor (LXR), in muscle, liver and SAT of Tfm mice compared to testosterone-replete animals whether with or without AR function leads to the hypothesis that testosterone may increase LXR signalling to exert some of its protective metabolic effects. LXRs are key transcriptional regulators of lipid and carbohydrate metabolism known to control molecular pathways including cholesterol efflux, glucose regulation, fatty acid synthesis and inflammation [46]. In parallel with testosterone-associated changes in LXR expression in the present study, we saw alterations in known LXR target genes: *Fasn*, *Apoe*, *Abca1*, *Lpl*, *Srebpf1*. Rather than inducing hepatic steatosis as with many LXR agonists, testosterone additionally protects against diet-induced hepatic lipid accumulation in this model [17]. Tfm mice also had reduced SAT and VAT expression of *Pparg* mRNA, indicating a potential mechanism by which testosterone deficiency may lead to metabolic dysregulation and adverse fat distribution. Additionally, Tfm mice displayed lower SAT *Ppara *(a master regulator of fatty acid oxidation) expression, suggesting that testosterone deficiency may further inhibit lipid regulation.

The present study indicates that testosterone may signal, at least in part, beyond its classical nuclear AR to modulate targets of lipid and glucose metabolism and that these actions are further differentially dependent on the target tissue. Whether the AR-independent effects in this study are via conversion to estradiol and subsequent activation of the oestrogen receptor (ER) was not addressed. We have previously shown, however, that testosterone has additional actions on hepatic and aortic lipid accumulation in Tfm mice even with aromatase inhibition and ER blockade [16, 17]. Further investigation is required to elucidate the AR-independent signalling mechanisms of testosterone action.

### Limitations

The present exploratory study is limited to target expression data, and while it indicates potential metabolic effects of testosterone it does not directly assess metabolic function. Lack of tissue prevented protein analysis of SAT and VAT due to the reduced amounts of protein recoverable from available adipose tissue. In addition, the Tfm mouse is a model of global AR dysfunction and severely reduced testosterone levels from birth, therefore we cannot rule out any developmental effects of these factors on tissues which may influence the pathogenesis of metabolic disorders. Whilst the testosterone injections produce levels within the normal range, diurnal patterns are absent and supraphysiological levels in the first few days are apparent with near-infraphysiologic levels towards the end of the interval [16]. Such administration may explain the influence of testosterone treatment on gene expression above and beyond that observed in wild-type controls. An additional orchidectomised XY littermate group receiving testosterone treatment would also allow us to control for pharmacological and dosing effects in animals with fully functional AR. These issues should be addressed in future studies.

## Conclusion

We present exploratory evidence that suggests testosterone is a metabolic hormone that differentially regulates the expression of key targets of lipid and glucose metabolism in a tissue-specific manner to potentially reduce fat deposition in pathologically relevant locations such as liver and the arterial tree. Indeed, as regional differences in the action of testosterone on subcutaneous and visceral adipose function are apparent, we hypothesise that low testosterone in the Tfm mouse leads to decreased lipid uptake and glucose utilisation in SAT resulting in its reduced capacity to act as a physiological ‘buffer’ in times of positive energy balance. This decreased ability to store excess lipid may then result in spillover into other tissues. Tfm mice have increased lipid accumulation in the aortic root and liver as early manifestations of atherosclerosis and hepatic steatosis. These effects are significantly reduced by testosterone replacement [17]. While this study adds support to the literature implicating testosterone as a metabolic hormone, by combining expressional data from multiple metabolic tissues with pathological evidence that testosterone protects against the development of hepatic steatosis and atherosclerosis, we now suggest a system-wide androgenic action to offer new mechanistic insight to the observed clinical benefit of testosterone in men with T2D and MetS.

## Footnotes

1 As previously published [16, 18, 20].

2 As previously published [15].
